# The impact of gingivitis reduction on lung function: a randomized trial under intensified oral hygiene

**DOI:** 10.1186/s13063-023-07135-0

**Published:** 2023-02-23

**Authors:** Jan Kühnisch, Tianyu Zhao, Randi J. Bertelsen, Rudolf A. Jörres, Dennis Nowak, Joachim Heinrich

**Affiliations:** 1 Department of Conservative Dentistry and Periodontology, University Hospital, Ludwig-Maximilians University Munich, Germany, Munich, Germany; 2 Institute and Clinic for Occupational, Social and Environmental Medicine, University Hospital, Ludwig-Maximilians University Munich, Germany, Munich, Germany; 3 Department of Clinical Science, University of Bergen, Bergen, Norway

**Keywords:** Gingivitis, Periodontitis, Oral health, Lung function, Spirometry, Microbiome

## Abstract

**Background:**

Periodontal disease and lung function impairment were found to be associated with low-grade systemic or local inflammation, and it might be that gingival/periodontal inflammation triggers lung function due to systemic inflammation or the transfer of oral bacteria or its components to the lung. A recent observational study in non-smoking subjects showed that lung volumes and flow rates were significantly reduced by 71–185 ml for severe gingivitis regardless of the adjustment for potential confounders. The result did not show any confounding by smoking, and the association between gingivitis and lower lung function was not modified by systemic inflammation. The designed interventional trial primarily aims to test the hypothesis that gingivitis reduction by optimized daily oral hygiene, professional tooth cleaning and antibacterial chlorhexidine (CHX)-containing mouth rinse improves lung function in terms of forced vital capacity (FVC) by at least 2%. The secondary objective will test the hypothesis that gingivitis reduction improves forced expiratory volume in 1 s (FEV1) and forced expiratory flow at 25–75% of the pulmonary volume (FEF25-75) by at least 2%. Furthermore, the influence of the oral microbiome will be taken into account.

**Methods:**

The study has to include 120 non-smoking subjects aged between 18 and 30 years with biofilm-induced gingivitis. The chosen “waiting control group design” will compare the immediate intervention group with the delayed intervention group, which serves as a control group. Dental and gingival status, lung function and oral microbiome will be recorded. The intensified preventive intervention—professional tooth cleaning, one-stage full-mouth disinfection with CHX and safeguarding an optimal daily oral hygiene by each subject—cannot be blinded, but the outcome measurement in terms of lung function tests is blind.

**Discussion:**

This proposed multidisciplinary study has several strengths. Only one previous intervention study with patients with severe periodontitis (mostly smokers) has been performed. It is novel to include non-smoking subjects with mild and potentially reversible oral inflammation. Furthermore, this research is innovative, because it includes evidence-based interventions for gingivitis reduction, standardized measures of the outcome on lung function and oral microbiome and combines expertise from dentistry, lung physiology, oral microbiology and epidemiology/statistical modelling.

**Trial registration:**

German Clinical Trial Register DRKS00028176. Registered on February 2022.

## Administrative information

Note: The numbers in curly brackets in this protocol refer to the SPIRIT checklist item numbers. The order of the items has been modified to group similar items (see http://www.equator-network.org/reporting-guidelines/spirit-2013-statement-defining-standard-protocol-items-for-clinical-trials/).

**Table:** 

Title {1}	The impact of gingivitis reduction on lung function in young adults: a randomized trial under intensified oral hygiene
Trial registration {2a and 2b}.	German Clinical Trial Register number DRKS00028176
Protocol version {3}	Not applicable.
Funding {4}	Application for funding by German Research Foundation (DFG) is ongoing.
Author details {5a}	Jan Kühnisch^1^, Tianyu Zhao^2^, Randi J. Bertelsen^3^, Rudolf A. Jörres^2^, Dennis Nowak^2^, Joachim Heinrich^2^ ^1^Department of Conservative Dentistry and Periodontology, University Hospital, Ludwig-Maximilians University Munich, Germany ^2^Institute and Clinic for Occupational, Social and Environmental Medicine, University Hospital of Ludwig-Maximilians University of Munich, Germany ^3^ Department of Clinical Science, University of Bergen, Bergen, Norway
Name and contact information for the trial sponsor {5b}	No trial sponsor.
Role of sponsor {5c}	Not applicable.

## Introduction

### Background and rationale {6a}

**Fig. 1:**
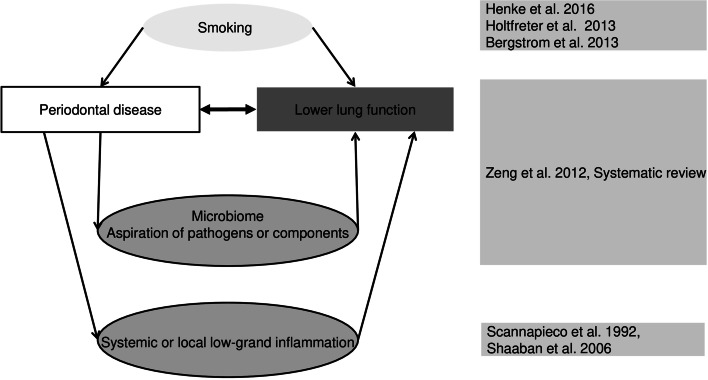
Epidemiological findings on the associations between periodontal diseases and COPD and lower lung function in adults and potential biological mechanisms

Lung function has been established several decades ago as a major predictor of increased mortality later in life (as reported by [1, 13]. Accordingly, recent epidemiological studies confirmed that even mild abnormalities in lung function within the clinically normal range were associated with increased risk for mortality as well as respiratory and cardiovascular abnormalities (e.g. [13, 65]. It is also important that reductions in lung function persist during the life course [1]. Based on the increasing body of evidence of low lung function linked to detrimental effects in later life, Faner and Agusti [14] concluded that simple spirometry testing at a young age should be implemented to identify high-risk individuals. They also concluded that the identification of determinants of lung function reduction in early life is highly warranted, as well as interventions against its further impairment. Among the potential determinants of low lung function, its association with periodontal diseases (PD), such as gingivitis or periodontitis, has been investigated in a number of studies. The latest systematic review [69] identified 14 observational studies and reported a pooled odds ratio of 2.08 (95% confidence interval 1.48, 2.91) for chronic obstructive pulmonary disease (COPD). Other authors [6, 11, 19, 21, 22, 45, 60] underlined the association of oral inflammation with adverse effects on the lung, although this finding could not be supported by all studies [4, 35, 56]. However, the most recent studies [17, 20–22, 44, 61] confirmed the overall association documented in a previous systematic review (see Fig. 1). These studies were mostly restricted to PD and COPD, but a systematic review also reported a significant association between PD and asthma [41]. Smoking is known as an important co-determinant for both PD and lung diseases, and consequently, studies on PD and COPD that adjusted for smoking showed a substantial attenuation of the association [4, 21, 22]. From the observational results, it is currently uncertain to which extent smoking, as a common risk factor, is sufficient to explain the association between PD and COPD.

**Fig. 2:**
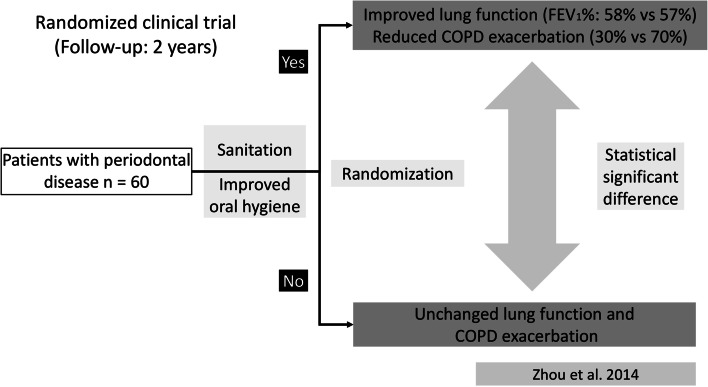
Interventional studies on lung function improvements and reduced COPD exacerbations in adults by reduction of periodontal diseases

**Fig. 3:**
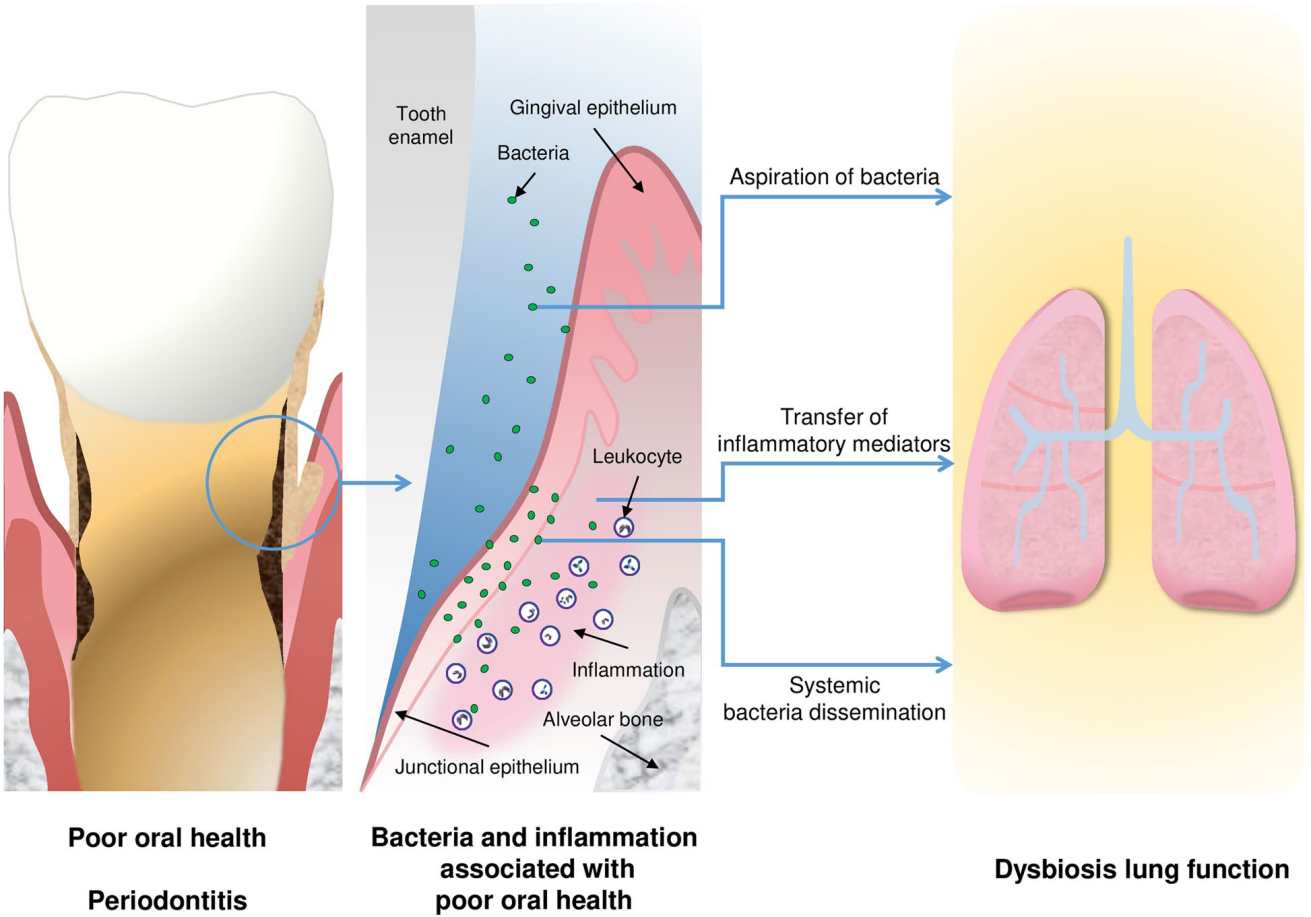
Potential biological mechanisms of how oral bacteria can affect the lung [18]

Therefore, the results of interventional studies, which control for smoking, are essential. Until now, three interventional trials have demonstrated positive effects of periodontal therapy in patients with COPD, among them two non-randomized trials [25, 57] and a randomized trial [70]. They found improved lung function and reduced exacerbation rates in COPD patients after oral hygiene instructions and periodontal treatment (see Fig. 2). However, these studies on oral inflammation and lung health were restricted to adults in advanced age, in whom smoking already acted as a common risk factor over a long time, with local inflammation and indirect effects due to systemic inflammation as possible underlying mechanisms [34, 53] (see also Figs. 1 and 3).

Regarding the details of the potential mechanisms, Linden et al. [34] stated that the inflammatory status of the airways might be affected by aspiration of dental plaque and/or haematogenous dissemination of inflammatory mediators and periodontal bacteria. In addition, a shared pathogenesis between both disorders via shared pathogens has been suggested [51]. PD is associated with low-grade systemic inflammation [16, 38, 46]. Since systemic low-grade inflammation is associated with both PD and lung function impairment [22, 52, 55, 62, 68], it may be that PD affects lung function also via systemic inflammation. As smoking is a strong determinant for both periodontal diseases [28, 30] and poor lung function, confounding has always to be taken into account, even in interventional trials. Thus, the association between periodontal diseases and lung function and/or COPD in the absence of smoking and smoking history is highly relevant. Based on this, we previously analysed the association between spirometric lung function and oral health indicators in a large group of more than 1000 non-smoking adolescents [20]. Spirometric lung volumes and flow rates were significantly reduced by 71–185 ml in the highest categories of gingivitis even after adjustment for other potential confounders in these non-smokers. This study supported the hypothesis that the reported association between poor periodontal health and low lung function is unlikely to be due to smoking alone. On the other hand, the study did not support the assumption that the association between gingivitis and lower lung function is caused by systemic inflammation, as far as indicated by the high sensitivity-C reactive protein.

It is worthwhile to mention that all presently available observational studies consistently showed lower forced expiratory volume in 1 s (FEV_1_) in the subgroups of poor oral health. However, these observations need to be interpreted with caution. Despite adjustment for several major co-factors such as smoking, socioeconomic status, chronic diseases, medication and acute infections during lung function testing, residual confounding cannot be ruled out in multifactorial processes. Moreover, all observational studies had a cross-sectional design, with the exception of one [21]. As a result of the data outlined above, there is a need for (a) interventional studies and (b) studies in younger age to validate the results obtained by Zhou et al. [70]. We combine both of these requirements in our planned study.

### Objectives {7}

The main objective is to test the hypothesis that gingivitis reduction via optimized daily oral hygiene and antibacterial chlorhexidine-containing (CHX) mouth rinsing improves lung function in terms of forced vital capacity (FVC), as an indicator of lung volume, by at least 2%. This allows interpreting the changes in lung function as direct or indirect consequences of changes in oral inflammation.

As a secondary objective, we test the hypothesis that gingivitis reduction by improved oral hygiene and CHX mouth rinsing improves forced expiratory volume in 1 s (FEV_1_) and forced expiratory flow rate at 25–75% of lung volume (FEF_25–75_), as indicators of airway obstruction, by at least 2%. Compared to the change in FVC, this will also allow to detect potential improvements regarding airway obstruction in terms of their ration FEV_1_/FVC. This is helpful for the interpretation of an improvement in lung function as a factor linked to a lower risk for obstructive airway disease.

### Trial design {8}

The design of this randomized clinical trial is a “waiting control group design”, which compares the immediate intervention group with the delayed intervention group. The latter one will serve as a control group. The intervention cannot be blinded, but the outcome measure (lung function tests) is blinded, because the technician does not know to which group (immediate or delayed intervention) the subject belongs to. Moreover, the absence of any intervention in subjects with gingivitis would be unethical, but an intervention delayed for 4 weeks seems acceptable.

## Methods: participants, interventions and outcomes

### Study setting {9}

This study will be executed in the academic outpatient dental clinic at the Ludwig-Maximilians-University of Munich.

### Eligibility criteria {10}

The study subjects should have an age between 18 and 30 years, because of the life period with peak achievements of lung function.

The following are the inclusion criteria:

- Non-smoking subjects with biofilm-induced gingivitis
- Participants must be vaccinated against COVID-19
- Negative test result for COVID-19 before each dental visit (needs to be modified in adherence to current regulations)

The following are the exclusion criteria:

- Active smoking
- Other gingival/ periodontal diseases [8]
- Having at least one tooth with a periodontal pocket depth and attachment loss > 4 mm
- Asthma, diabetes and other chronic diseases, and anti-inflammatory treatment for chronic diseases
- Pregnancy
- Long COVID-19
- Not able/willing to provide informed consent
- Not available for the period of repeated examinations

The recruiting will start with the pre-selections of subjects with occasional gum bleeding. These subjects are screened for further eligibility for the intervention study. The continuous recruitment will address primarily patients of dental school and university students.

### Who will take informed consent? {26a}

The informed consent will be obtained by the recruiting dentists.

### Additional consent provisions for collection and use of participant data and biological specimens {26b}

On the consent form, participants will be asked if they agree to the use of their data should they choose to withdraw from the trial. Participants will also be asked for permission for the research team to share relevant data with people from the cooperating universities. This trial does not involve collecting biological specimens for storage.

### Interventions

#### Explanation for the choice of comparators {6b}

Safeguarding good and lifelong oral hygiene is a prerequisite for preserving dental and oral health. During the last decades, many associations between oral health—especially periodontal health—and general medical diseases, e.g. diabetes, cardiovascular diseases, adipositas/obesity, chronic kidney disease or chronic obstructive pulmonary diseases, have been described [7, 28, 29, 31, 43, 54]. Many studies followed a wide variety of scientific questions but mainly included older adult populations. Interestingly, there is limited information available for younger groups which may enter the initiation phase of both periodontal and general diseases. Available studies have shown that potential associations between periodontal health and potential risk factors became detectable also in young populations. Here, Pitchika et al. [46] have shown that elevated high-sensitivity C-reactive protein blood levels were positively associated with gingival health, smoking habits and overweight/obesity. On the basis of this finding, it can be concluded that other known associations in adults might be probable transferable to a younger age as well.

#### Intervention description {11a}

##### Intervention 1

The association between microbial dental biofilm and caries is known for decades [18, 32, 39]. Furthermore, there is clear evidence that the (non-)presence of dental biofilm triggers the initiation, persistence, progression or healing of dental biofilm-induced gingivitis which may be later progressed into periodontitis [8, 9, 32, 63]. Therefore, the daily control of the dental biofilm by each individual is essential over the lifetime and needs to be considered in clinical studies due to many reasons as well. In the present clinical study, oral hygiene instructions are needed to (1) provide the essential knowledge to safeguard dental well-being as best as possible which goes hand in hand with the scientific aim (2) to standardize the daily oral hygiene routines between all study subjects over the whole study period. Therefore, intensified oral hygiene instructions which are based on recently published evidence-based recommendations by the German Society of Dentistry [12] are part of the “intervention 1” phase and include the following preventive measures:

- Motivational interviewing, individualized demonstration and instruction for optimal, systematic and regular oral hygiene using a vertical brushing technique (e.g. BASS technique).
- Recommendation to use fluoridated toothpaste (fluoride content 1000–1450 ppm).
- Instruction for using dental floss due to the fact that proximal niches cannot be cleaned by tooth brushing alone. Worthington et al. [67] concluded in their most recently published Cochrane review that the use of dental floss or interdental brushes in addition to tooth brushing may reduce gingivitis or plaque, or both.

To ensure an ongoing acceptance and the motivated use of basic oral hygiene measures, all participants will be repeatedly informed during the study visits about the benefits of simple and basic preventive measures.

##### Intervention 2

The professionally administered supra- and subgingival biofilm control goes hand-in-hand with the removal of periopathogens improves gingival inflammation [10] and is, therefore, an essential part of the 2nd intervention phase. This procedure includes the removal of calculus using hand instruments, e.g. scalers, and/or ultra-sonic instruments. In the second step, all teeth will be systematically cleaned using rotating toothbrushes with a low-abrasive polishing paste. In the last step, all teeth will be fluoridated using a varnish (Fluor Protector, Vivadent, Schaan, Lichtenstein). As pointed out before, each subject will be informed about their oral health status, and appropriate oral hygiene instructions will be provided by the professional dental team.

In addition to the professional tooth cleaning a full-mouth disinfection using a non-alcoholic, CHX-containing mouth rinse is included in the study protocol which has to be applied twice a day over a period of 14 days. Recent studies [3, 15, 49] showed short-term clinical benefits of one-stage full-mouth disinfection, when compared to the standard treatment strategy of consecutive tooth cleaning alone. Here, the mouth rinse may help to enable proper disinfection of the remaining intraoral niches which could not be reached by the dental instruments especially in patients suffering from periodontitis [50]. With respect to gingivitis patients, van Strydonck et al. [64] analysed the available literature in a systematic review and concluded that CHX mouth rinses together with oral hygiene instruction provided a significant reduction in plaque and gingivitis scores. In another more recently published Cochrane review by James et al. [23], the effectiveness of CHX mouth rinse used as an adjunct to mechanical oral hygiene procedures for the control of gingivitis was assessed in more detail. The authors concluded that there is high-quality evidence of a substantial, but short-term reduction in dental plaque formation when CHX mouth rinse was used as an adjunct to mechanical oral hygiene. In case of gingivitis, the data were insufficient to determine the reduction in gingivitis in association with CHX mouth rinse. Nevertheless, after 4 to 6 weeks of use, CHX mouth rinse reduced gingivitis by an odds ratio of 0.21 (95%CI 0.11 to 0.31) compared to placebo, control or no mouth rinse. There is no evidence that one concentration of CHX mouth rinse (0.1%/0.12%/0.2%) is more effective than another [23]. Nevertheless, the CHX effect on the biofilm is dose-dependent, and the optimum CHX dose is considered to be 20 mg twice daily [36] which represents an equivalent of 10 ml of 0.2% CHX mouth rinse [24]. A rinse time of 30 s appears to be effective and acceptable [24].

Rinsing with CHX mouth rinse for 4 weeks or longer may cause harmful extrinsic tooth staining. Therefore, all study subjects will have access to an additional tooth cleaning after finishing the study protocol.

#### Criteria for discontinuing or modifying allocated interventions {11b}

It is not planned to discontinue or modify the intervention for trial participants.

#### Strategies to improve adherence to interventions {11c}

A key issue in maintaining the adherence of study participants is to explain the importance of the trial, to illustrate the study protocol and why it is important to adhere to the scheduled appointments and the given oral hygiene recommendation consistently. Furthermore, the acceptance of basic oral hygiene measures in all participants will be ensured by remotivation. With respect to the short recall intervals, no phone checks were planned.

#### Relevant concomitant care permitted or prohibited during the trial {11d}

There are no restrictions regarding concomitant medical or dental care during study participation.

#### Provisions for post-trial care {30}

The study protocol includes only established preventive dental interventions. Therefore, post-trial access to products or agents which are commercially non-available is not applicable for this trial.

### Outcomes {12}

The primary outcome is the pre- and post-bronchodilation lung function testing with the recording of the following clinical variables:

- Forced vital capacity (FVC)
- Forced expiratory volume in 1 s (FEV_1_)
- Forced expiratory flow at 25–75% of the pulmonary volume (FEF_25–75_)

### Lung function testing by spirometry

Spirometry prior to bronchodilation will be performed in line with ATS/ERS recommendations [40]. Subjects will sit while wearing nose clips, and the EasyOne Worldspirometer (ndd, Zurich, Switzerland) will be used to obtain flow-volume curves. To obtain optimal flow-volume curves, the participants perform at least three, but no more than eight, manoeuvres under the guidance of specifically trained and experienced examiners. All tests will be visually inspected by experienced physicians according to the ATS/ERS acceptability criteria [40]. Spirometric indices will be taken from the manoeuvre with the largest sum of forced expiratory volume in 1 s (FEV_1_) and forced vital capacity (FVC). Further parameters evaluated will be the ratio of FEV_1_ and FVC (FEV_1_/FVC), and the mean flow rate between 25 and 75% of FVC (FEF_25–75_). Standardized *z*-scores of the lung function parameters FEV_1_, FVC, FEV_1_/FVC and FEF_25–75_ will be calculated based on the reference equations for spirometry from the Global Lung Initiative (GLI—http://www.ers-education.org/guidelines/global-lung-function-initiative.aspx, [48]. After completion of baseline spirometry, subjects will inhale bronchodilator medication according to the ATS/ERS recommendations [40]. Two puffs (each 100 µg) of salbutamol will be delivered into a spacer (volumatic) by a metered-dose inhaler, and the participant will be asked to take five slow and deep breaths over 5–10 s for optimal deposition. Spirometry will be repeated 15 min after salbutamol inhalation. This analysis uses post-bronchodilator lung function data. A positive response is defined as an increase by > 12% and > 200 ml in FEV_1_ and/or FVC post-bronchodilation compared to pre-bronchodilation, according to ATS/ERS standards [40].

Specific attention will be paid to the requirement to obtain very precise and reliable lung function data. In addition to strictly following ATS/ERS recommendations, we will reach the goal of high-quality lung function testing by experienced and well-trained study nurses, the additional visual control by a pulmonologist and the repetition of tests for each subject. There is long-standing, well-documented expertise on lung function assessment in the investigators involved in this study.

### Biosampling and measurements of the microbiome

The secondary outcome is considered the bacterial composition. Both saliva and gingival fluid will be collected from the participants. Gingival fluid between the teeth will be collected with sterile paper points at 6 per-protocol predetermined sites in the lower jaw (mesial and distal at the “Ramfjord” teeth 41, 44 and 36) and 6 sites in the upper jaw (mesial and distal at the “Ramfjord” teeth 21, 24, 16). The samples will be frozen directly after collection, in separate vials for the upper and lower jaw samples. Saliva will be collected as well. Subjects are asked to chew for 1 min on a paraffin gum.

### Participant timeline {13}

**Fig. 4:**
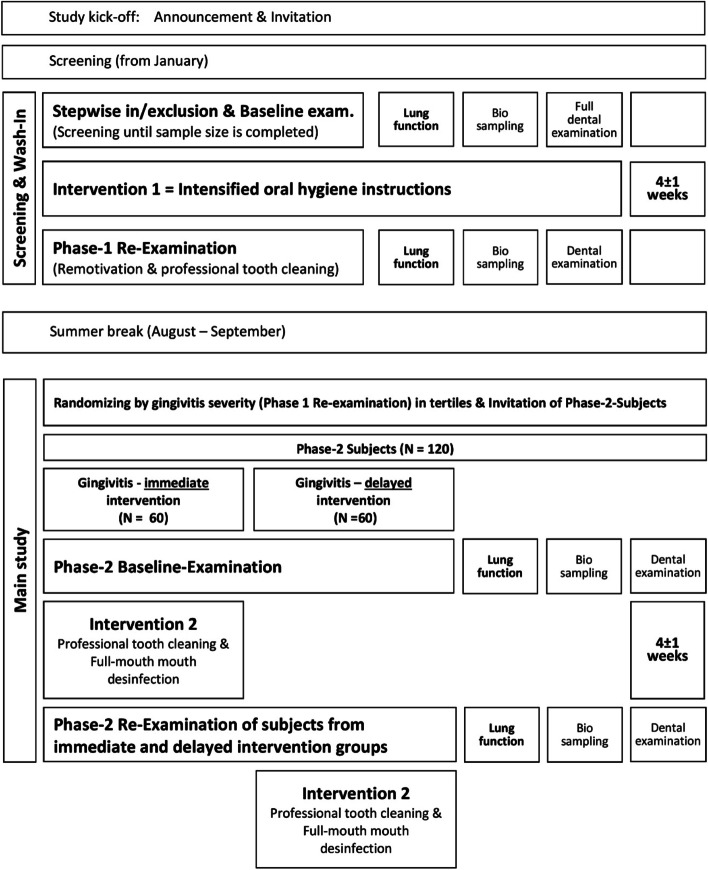
Flowchart of the study

The time schedule of enrolment, interventions (including any run-ins and washouts), assessments and visits for participants is illustrated in the following schematic diagram (Fig. 4). From our clinical experiences, we expect a cohort of individuals with mostly good to moderate oral hygiene. To address this heterogeneity appropriately, we provide a wash-in phase with oral hygiene instructions to equalize oral health in the study population. Signs of gingivitis are mostly reversible within 2 to 4 weeks when oral hygiene is improved. Following an interim data analysis, the main study protocol is carried out.

### Sample size {14}

The calculation of the power of this study is complicated by the fact that only limited information on changes in lung function is available. The only published trial on oral sanitation and its impact on lung function included 2 × 30 subjects with severe periodontal diseases and an average age of 65.7 years. The authors reported an improvement in FEV_1_ by 3–4%predicted [70]. Whether the effect size of this study can be used to derive a reliable power calculation for our study seems questionable, mainly because of the advanced age of the study subjects and the progression of periodontal diseases in this age group. We also cannot infer whether we should expect weaker or stronger effects in our young study group with only mildconfirmed the overall association documented in a previo gingivitis, compared to the intervention in the Chinese population of older subjects. One might speculate that in our study effects would be stronger, due to a mild inflammatory status of younger subjects with gingivitis, which might be easier attenuated or abolished compared to a long-lasting inflammation accompanied by persistent structural changes of the periodont and possibly the lung. Such irreversible changes in the lung are less likely in our study, given the young age of the study subjects. As far as reversibility is concerned, the expected effect size on the lung might be even larger compared to the Chinese interventional study, given the same degree of oral inflammation. These considerations show that the power analysis has to be largely based on plausibility arguments and only partially on available data. Other types of interventions might be useful to estimate the time scale needed. For example, a complex rehabilitation programme comprising physical activity, dietary intervention and strongly monitored medication resulted in short-term improvements of FEV_1_ by 8% predicted in asthmatic patients aged 4–19 years, within an intervention period of less than 4 weeks [5]. Thus, our follow-up period might be sufficient.

Observational studies could also be used for power calculation. The few studies on oral health and lung function consistently found lower lung function in subjects with periodontitis or gingivitis. A cross-sectional study in 1463 German adults aged 25 to 86 years showed FEV_1_ values lower by 5% predicted in subjects with clinical attachment loss of more than 50%, after adjustment for several co-factors. The non-smokers showed even further lowered FEV_1_, by more than 10% predicted [22]. Similarly, a prospective follow-up study of 206 subjects (mean age 42 years) found 10% lower FEV_1_ in subjects with periodontitis [21]. The third observational study of more than 650 subjects aged 50 + years from Scandinavia reported FEV_1_ values lower by 5% predicted for subjects with poor oral health [44]. The only available population-based study in younger age (15 years) showed FEV_1_ to be lower by about 20% predicted in the approximately 10% of adolescents with the highest SBI or CPI [20]. Active smoking was uncommon at this age, and the few active smokers were excluded from this analysis. Noteworthy, all five observational studies consistently showed lower FEV_1_ in subgroups of poor oral health. Despite this, some caution is needed in the interpretation. Although some of these studies included co-variates such as smoking, socioeconomic status, chronic diseases and medication, acute infections during lung function testing and early life factors, residual confounding cannot be ruled out in view of the multiplicity of potential influencing factors. Lung function has many determinants and is varying over time. This is the reason for further interventional study in subjects of younger age. Based on the critical evaluation of the study findings on oral health and lung function, we prefer a conservative approach for our power calculation.

The power analysis focuses on the second part of the study, in which two different treatments are realized, whereby in both arms assessments before and after treatment are performed (Fig. 4). As the control treatment may be expected to be associated with a positive change, although smaller than in the target treatment group, the assumed effect is taken to be greater than zero. Thus, relative effects of m1 = 2 and of m2 = 1 are assumed for the target treatment and the control treatment group, respectively, in units that can be adapted to those of the outcome measures. There are no sufficient data on the (longitudinal) variation of measured values due to either methodological or biological sources. The power analysis is performed with an assumed variance of 1.5 for each assessment, in units identical to those of the effects and corresponding to a standard deviation (SD, square root of variance) greater than 1 but less than 1.5, which we consider as a reasonable value. Assuming that the measurement errors are independent in the pre and post-assessments, the variances add up for the pre-post differences, resulting in a total variance of each of the two differences of 3, i.e. an assumed standard deviation of about 1.7 (square root of 3) for each difference. Thus, we have two groups with expected effects of 2 or 1, both with standard deviations of about 1.7. This seems a reasonable basis for analysis, whereby the mean differences observed in the two treatment arms have to be compared in a standard 2-means comparison based on normal distributions. The comparison is performed two-sided in order to be on the safe side, although the hypothesis is that treatment arm 1 is more effective than treatment arm 2. Requiring a power of 90% (beta = 0.1, error of the second kind) at a significance level of 5% (alpha = 0.05, error of the first kind), the assumed parameter values result in sample sizes of *n* = 61 for each of the two treatment groups. When assuming variances of either 1 or 2 instead of 1.5 for each of the assessments, the required group sizes would be *n* = 42 or *n* = 84, respectively. In the latter case, a sample size of *n* = 63 in each group would still be sufficient for alpha = 0.05 at a power of 80% (beta = 0.2), which is often considered as sufficient in clinical trials. Thus, based on reasonable assumptions and conventional criteria, sample sizes of about 60 subjects per group should be adequate to perform this clinical intervention study successfully and to obtain a conclusive result.

### Recruitment {15}

The recruiting will start with the pre-selections of subjects with occasional gingival bleeding. These subjects are screened for further eligibility for the intervention study. The continuous recruitment will address primarily patients of dental school and university students.

### Assignment of interventions: allocation

#### Sequence generation {16a}

The main aim of the randomized allocation process will be to *equalize* both intervention groups on basis of the initially documented oral health data under the inclusion of caries and gingival/ periodontal status [2, 9, 26, 27, 37, 42, 47, 58, 66]. This requires an analysis of dental screening and re-examination data. On basis of the gingival health data, a randomized allocation list will be established.

#### Concealment mechanism {16b}

In principle, all participants will receive the same preventive measures owing to ethical reasons. Following the allocation of all subjects to one of the intervention groups, each participant will be informed by telephone about the appointment for the phase 2 baseline examination. As the protocol for the two groups—immediate and delayed intervention—cannot be blinded for the study team and participants, no efforts will be made to conceal this information.

#### Implementation {16c}

The generation of the allocation will be done by the data management group independently from clinicians.

### Assignment of interventions: blinding

#### Who will be blinded {17a}

The clinical research group will be blinded against the data analysts. Trial participants cannot be blinded.

#### Procedure for unblinding if needed {17b}

The design is open-label with only outcome assessors being blinded so unblinding will not occur.

### Data collection and management

#### Plans for assessment and collection of outcomes {18a}

The clinical investigation plan is illustrated in Fig. 4. The collection of the above-mentioned outcome data includes standardized, reliable and widely accepted methods and indices only.

#### Plans to promote participant retention and complete follow-up {18b}

An important issue in maintaining the retention of study participants is to explain the importance of prevention for individual dental health and to illustrate potential effects on medical health which are investigated in the present trial. In addition, the acceptance of basic oral hygiene measures in all participants will be ensured by remotivation.

#### Data management {19}

Data management includes checks for the plausibility of all dental, spirometry data and microbiome data.

#### Confidentiality {27}

Typically, the recording of all clinical data will be handled using paper-based case report forms. The data will be digitalized immediately to make a subsequent data analysis available. All data will be collected and stored on secured university-based computers to protect confidentiality before, during and after the trial. The statutory periods of data retention will be safeguarded.

#### Plans for collection, laboratory evaluation and storage of biological specimens for genetic or molecular analysis in this trial/future use {33}

Saliva and/or gingival samples collected during each of the follow-ups will be analysed with high-throughput sequencing techniques to characterize the bacteria that are present and to determine bacterial diversity. Bacterial DNA from the 16S rRNA gene will be isolated and the V3–V4 region of the 16S rRNA amplified and sequenced by the Illumina® MiSeq platform. The resulting sequences will be assigned according to taxonomy by the Human Oral Microbiome Database (www.homd.org), which contains information of all known cultivated and sequenced bacteria in the human oral cavity. It will also be possible to apply quantitative PCR (count number of sequences) for bacteria that are identified in the output of the high-sequencing techniques to be of particular importance for gingivitis status and lung health. This will enable us to get a better estimate of the relative contribution of these bacteria in the gingival and/or saliva samples.

Quality control and advanced upstream statistical analyses will be performed in QIIME 2 and R. We will model the differential abundance of bacteria in the samples, presented by diversity indices, such as the Shannon index with the QIIME software. The Analysis of composition of microbiomes with bias correction (ANCOM-BC) methodology [33] will be used to model the differential abundance in the oral microbiome by gingivitis status and for changes in bacterial composition for repeated saliva and/or gingiva fluid samples. The false discovery rate (FDR) will be controlled at the 5% level [59] to adjust for multiple testing.

## Statistical methods

### Statistical methods for primary and secondary outcomes {20a}

Processing and evaluation of data include checks for the plausibility of spirometry data and microbiome data. Moreover, descriptive data analysis will be used to check the plausibility of the distribution of data. For evaluating contextual and compositional determinants, low and high levels of outcome data will be used. In addition, potential determinants for each of the outcomes will be explored by linear regression modelling and will be checked for potential adjustment. Within these approaches, different methods of confounder adjustment (general fixed and random mixed models) will be applied for different association patterns of determinants and risk factors and for risk factor-outcome relationships in cases of clustered risk factors. For repeatedly evaluated measures throughout regression models for longitudinal data, such as the generalized estimating equations (GEE) approach may be applied.

To account for the control arm of this interventional study, multilevel statistical models can be used that integrate information on risk factor-outcome relationships on two complementary levels of intervention and non-interventional group. Such multilevel models can take confounding variables fully into account and are well-suited for studying or identifying specific group effects.

### Interim analyses {21b}

The study protocol requires interim data analyses after the screening and wash-in phase. This analysis will be carried out by the data analysis group and has to be understood as a prerequisite for forming both intervention groups.

### Methods for additional analyses (e.g. subgroup analyses) {20b}

Descriptive and explorative analyses for the planned interventions are planned.

### Methods in analysis to handle protocol non-adherence and any statistical methods to handle missing data {20c}

In case of non-adherence, each individual will be contacted immediately and re-invited to follow the intended timeline. If individuals are unable to proceed with study participation, the corresponding data will be excluded from the phase 1 or phase 2 dataset (lost to follow-up).

### Plans to give access to the full protocol, participant-level data and statistical code {31c}

The datasets analysed during the current study and statistical code are available from the corresponding author upon reasonable request, as is the full protocol.

### Oversight and monitoring

#### Composition of the coordinating centre and trial steering committee {5d}

The role of the trial steering committee is to provide overall supervision for the trial and provide advice to the trial management group at both study centres, data analysts and the sponsor. The administrators will review and implement the study protocol. Furthermore, they supervise the trial management group when it is initiated. The latter one is responsible for the day-to-day running and management of the trial and meets regularly.

#### Composition of the data monitoring committee, its role and reporting structure {21a}

This study uses approved widely used preventive agents, e.g. CHX, or products, e.g. tooth polishing paste, only. Therefore, the potential risk of adverse events is very low and justifies the decision that a data monitoring committee (DMC) is not needed.

#### Adverse event reporting and harms {22}

This study uses only approved widely used preventive agents, e.g. CHX, and therefore, the risk of adverse events can be assessed as very low and justifies the decision that a systematic registration of adverse events is not included in the trial design.

#### Frequency and plans for auditing trial conduct {23}

With respect to the non-availability of an external sponsor, it is not planned to engage an external trial auditor, and therefore, the study group is responsible by itself to ensure that all information is credible, i.e. that all scientific data has been generated, collected and processed are methodologically correct. Regular group meetings will be used to monitor the study quality as well as to optimize procedures in case of problems Furthermore, the study group respect laws and regulations.

#### Plans for communicating important protocol amendments to relevant parties (e.g. trial participants, ethical committees) {25}

The Ethics Committee of the Medical Faculty of the Ludwig-Maximilians University of Munich reviewed and approved the study concept (project number 019–998).

#### Dissemination plans {31a}

Since the results of an interventional study are closer to derive recommendations compared to observational studies, a detailed dissemination plan of study findings will be developed. This plan goes beyond publication in high-ranked peer-review journals. It includes oral presentations at national and international congresses of lung physicians and dentists, the inclusion of medical and dental societies and the use of their information channels, and the dissemination via public media and patient organizations.

## Discussion

This proposed study has several strengths: (1) It is novel, because it includes subjects with mild (and potentially reversible) oral inflammation. (2) It is innovative, because it includes oral sanitization with lung function testing and measurements of the oral microbiome. (3) It has a high public health relevance, because this study examines the potential improvement of lung function in a period of maximum achieved lung function or at least before a major decline of lung function started and has the potential to develop a strategy to counteract against lung diseases in later life. (4) It might add a piece of causality, because of the interventional aspects and mediating effects by the changes of the microbiome. (5) It is a multidisciplinary research which combines expertise on lung physiology and lung function testing with oral health and oral microbiome and epidemiological skills and statistically modelling. (6) It is a cutting-edge project, because it brings together international experts in the field from Norway (PI: Randi J. Bertelsen), with the potential to extend this interventional study to other countries. (7) It goes beyond a simple observation and hypothesis generation, because this interventional study is a proof of principle.

However, this study has also some limitations. The major limitation is related to the uncertainty about the results, because of the high novelty and the innovative characteristics of this proposed study. Due to the high methodological experience of the study group, the strong focus of an interventional study and the strong design, we believe to provide a sound answer to the underlying research question regardless of whether we could prove the primary hypothesis. (8) Finally, this trial also might have clinical implications, although the effect size of lung function changes may seem small. The power calculation of the study is based on assumed changes of 2% for FEV_1_ and FEF_25–75_ during the time of the trial. According to the available data, this is a justified and realistic assumption of short-term changes in lung function due to the oral sanitation programme. This assumed change can be considered as a conservative estimate, since real effects are more likely to be higher than smaller. From a clinical perspective, a 2% improvement in lung function per se is certainly small, but one has to put this into a lifelong scenario, considering the natural decline of lung function over time. If the improvement is not a one-time event but, at least in part, a reduction in the annual rate of decline, the resulting difference in lung function after a number of years might very well matter, particularly.

## Trial status

At present, the study protocol is fully developed and an application for funding by the German Research Foundation (Deutsche Forschungsgemeinschaft, DFG) is ongoing. It is planned to begin recruitment in 2022/23.

## Data Availability

It is possible to make the full protocol, participant-level dataset and statistical code available on the basis of a reasonable request. Patient and public involvement: There was no public or patient involvement in the design of the protocol.

## Abbreviations

CHX: Chlorhexidine gluconate
COPD: Chronic obstructive pulmonary disease
FVC: Forced vital capacity
FEV_1_: Forced expiratory volume in 1 s
FEF_25__–__75_: Forced expiratory flow at 25–75% of the pulmonary volume
PD: Periodontal diseases
